# Crystal structure of (4-chloro­phen­yl)(4-methyl­piperidin-1-yl)methanone

**DOI:** 10.1107/S2056989020001930

**Published:** 2020-03-13

**Authors:** J. Srividya, D. Reuben Jonathan, B. K. Revathi, M. Divya Bharathi, G. Anbalagan

**Affiliations:** a PG and Research Department of Physics, Queen Mary’s College, Affiliated to University of Madras, Chennai-4, Tamilnadu, India; b Department of Chemistry, Madras Christian College, Affiliated to University of Madras, Chennai-59, Tamilnadu, India; c Department of Physics, Madras Christian College, Affiliated to University of Madras, Chennai-59, Tamilnadu, India; d PG and Research Department of Physics, Presidency College, Affiliated to University of Madras, Chennai-5, Tamilnadu, India; e Department of Nuclear Physics, University of Madras, Chennai-25, Tamilnadu, India

**Keywords:** crystal structure, piperidine, piperidin-1-yl, Hirshfeld surface

## Abstract

The title compound, C_13_H_16_ClNO, contains a methyl­piperidine ring in the stable chair conformation. The mean plane of the twisted piperidine ring subtends a dihedral angle of 39.89 (7)° with that of the benzene ring. In the crystal, weak C—H⋯O inter­actions link the mol­ecules along the *a*-axis direction to form infinite mol­ecular chains. H⋯H inter­atomic inter­actions, C—H⋯O inter­molecular inter­actions and weak dispersive forces stabilize mol­ecular packing and form a supra­molecular network, as established by Hirshfeld surface analysis.

## Abstract 2

The title compound forms triclinic crystals in which the methyl­piperidine ring is in its stable chair conformation. The dihedral angle between the mean planes of the benzene ring and that of the twisted piperidine ring is 39.89 (7)°. In the crystal, weak C—H⋯O inter­actions link the mol­ecules into infinite chains along the *a-*axis direction.

## Chemical context   

The structures of a wide variety of heterocyclic derivatives have been analysed for their pharma-potentiality over the past three decades (Katritzky, 2010 ▸). Among them, derivatives of the six-membered heterocyclic base piperidine have proven to be successful pharmacophores. Naturally existing in abundance, alkaloids of substituted piperidine compounds exhibit a wide range of biological activities (Yunusov & Azimova, 2013 ▸). Anti-convulsant (Santucci *et al.*, 1986 ▸), anti-tumor, anti-bacterial (Vinaya *et al.*, 2009 ▸), anti-viral (Abdel-Aziza *et al.*, 2010 ▸), anti-fungal (Rafiq *et al.*, 2013 ▸) and plasma triglyceride-lowering (Uto *et al.*, 2010 ▸) activities, along with their antagon­ist activity as anti-HIV-1 agents (Imamura *et al.*, 2005 ▸) are deserving of mention. Piperidin-1-yl derivatives have proven vital in the field of neuropsychosis due to their potent biological activity. They act as either central nervous system (CNS) depressants or as stimuli, based on dosage levels (Ramalingan *et al.*, 2004 ▸), and also show anti-tubercular (Patel *et al.*, 2011 ▸), anti-cancer (Lefranc *et al.*, 2013 ▸), anti-tumor (da Silveira *et al.*, 2017 ▸) and, in particular, anti-leukemic (Vinaya *et al.*, 2011 ▸) activities. One such active piperidin-1-yl derivative is the title compound, (4-chloro­phen­yl)(4-methyl piperidin-1-yl)methanone.
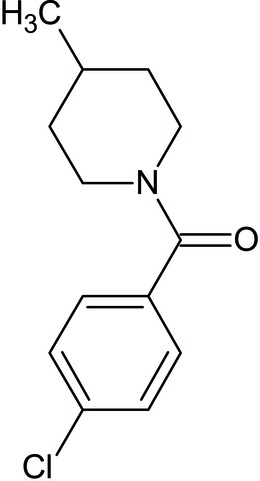


## Structural commentary   

**Figure 1:**
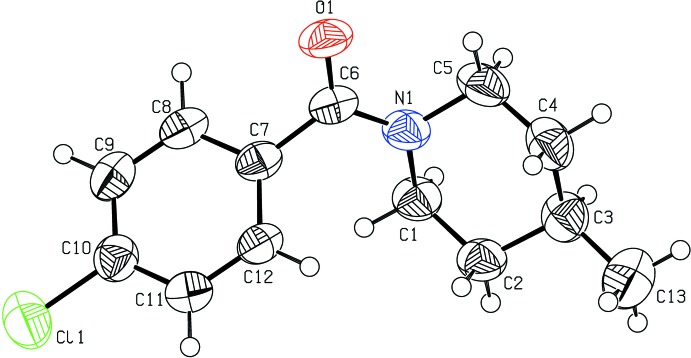
The mol­ecular structure of the title compound showing the atom-labelling scheme with displacement ellipsoids drawn at the 30% probability level.

The mol­ecular structure of the title compound, which features a chloro­benzene ring and a methyl­piperidine ring, is shown in Fig. 1 ▸. The C—N distances [1.343 (3)–1.462 (3) Å], C=O distance [1.233 (3) Å] and all other primary bond lengths along with bond angles are well within the range reported for similar structures (Prathebha *et al.*, 2015 ▸). The ring puckering parameters [*q*_2_ = 0.005 (3), *q*_3_ = −0.551 (3), *Q*_T_ = 0.551 (3) Å, φ_2_ = 203 (32)° and θ = 180.0 (3)°] confirm that the piperidine ring adopts a chair conformation. The C1—N1—C6—O1 and O1—C6—C7—C8 torsion angles are −167.4 (2) and 50.7 (3)°, respectively. The C1—C2—C3—C13 torsion angle [177.7 (2)°] reveals the anti-periplanar (+ap) orientation of the methyl group with respect to the piperidine ring.

## Supra­molecular features   

**Figure 2:**
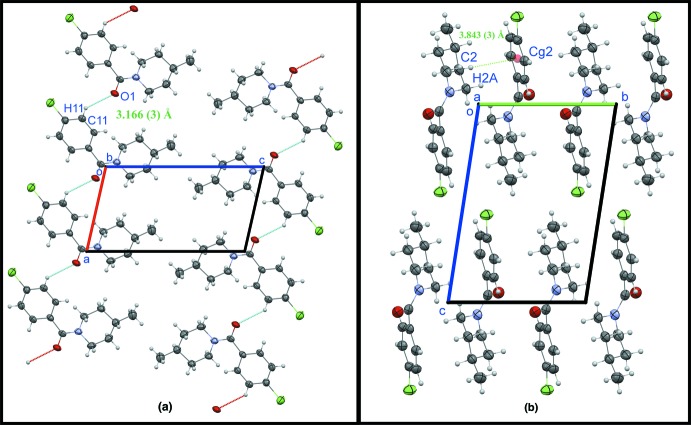
(*a*) The mol­ecular packing viewed perpendicular to the *ac* plane, showing the formation of chains along the *a* axis. Dotted lines indicate weak C—H⋯O inter­actions. (*b*) The crystal packing viewed along the *a* axis, showing the weak C—H⋯π close contacts (green dotted line).

**Figure 3:**
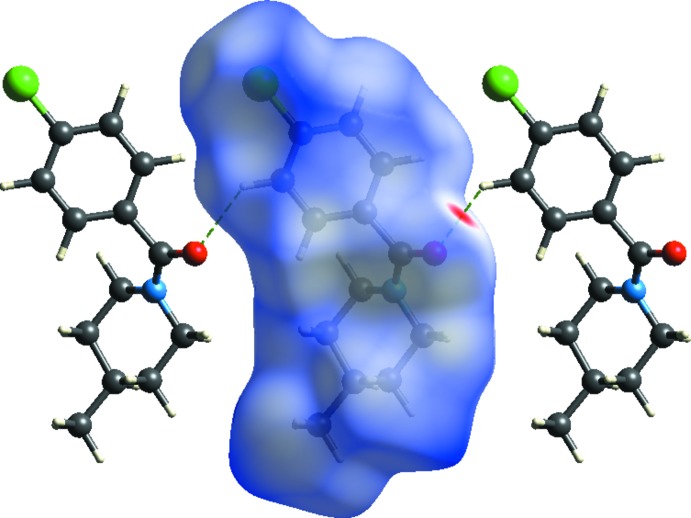
Hirshfeld surfaces mapped over *d*_norm_ showing weak C—H⋯O inter­actions on either side of the mol­ecule.

**Figure 4:**
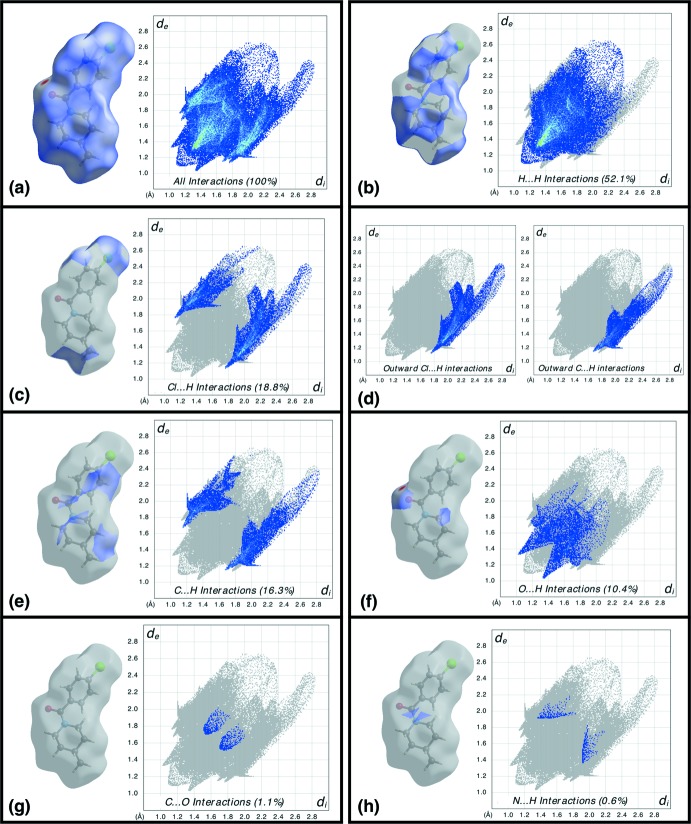
Two-dimensional fingerprint plots illustrating the percentage contributions of contacts within the crystal: (*a*) all inter­molecular inter­actions, (*b*) H⋯H contacts, (*c*) H⋯Cl/Cl⋯H contacts, (*d*) Outward Cl⋯H and C⋯H inter­actions, (*e*) H⋯C/C⋯H contacts, (*f*) O⋯H/H⋯O contacts, (*g*) C⋯O/O⋯C contacts and (*h*) N⋯H/H⋯N contacts. The Hirshfeld surfaces mapped over *d*_norm_ are displayed in grey, with the relevant surface patches associated with the specific contacts highlighted in colour.

In the crystal, weak C11—H11⋯O1 inter­actions link translation-related mol­ecules (*x* − 1, *y*, *z*), forming chains parallel to the *a* axis (Table 1 ▸, Fig. 2 ▸ *a*). Weak C—H⋯π close contacts between H5*A* and the benzene ring of an adjacent (1 − *x*, −*y*, −*z*) mol­ecule provide linkage between inversion-related (*i.e*., head-to-tail) chains (Table 1 ▸, Fig. 2 ▸ *b*). Analysis of the Hirshfeld surface (Spackman *et al.*, 2009 ▸) and the associated two-dimensional fingerprint plots (McKinnon *et al.*, 2007 ▸) were performed with *CrystalExplorer 17* (Turner *et al.*, 2017 ▸). Hirshfeld surfaces mapped over *d*_norm_, were generated using *TONTO* (Jayatilaka *et al.*, 2005 ▸) computations with B3LYP/6-31G(d,p) basis sets (Fig. 3 ▸). Among the major non-bonding inter­actions (Fig. 4 ▸), H⋯H contacts have the highest percentage contribution of 52.1%, followed by Cl⋯H/H⋯Cl (18.8%), C⋯H/H⋯C (16.3%), O⋯H/H⋯O (10.4%) and C⋯O/O⋯C (1.1%) inter­actions. The electrostatic and the polarization energies observed among the mol­ecules are compensated by the repulsive components, while the C—H⋯O inter­actions along with van der Waals dispersive forces contribute to form the supra­molecular network.

## Database survey   

The Cambridge Structural Database (version 5.40, Nov. 2018; Groom *et al.*, 2016 ▸) includes various structural analogues of substituted piperidin-1-yl compounds, which include EYIXIT (Schmittel *et al.*, 2004 ▸), AFETUB (Rao *et al.*, 2007 ▸), IJUZAP (Betz *et al.*, 2011 ▸), NIPCAS (Prathebha *et al.*, 2013 ▸), QUTGOD (Revathi *et al.*, 2015*c* ▸), NUKDUU (Revathi *et al.*, 2015*d* ▸), BEBFEW (Mohamooda Sumaya *et al.*, 2017 ▸), GUVXAY (Revathi *et al.*, 2015*a* ▸) and LUPDUX (Revathi *et al.*, 2015*b* ▸).

## Synthesis and crystallization   

**Figure 5:**
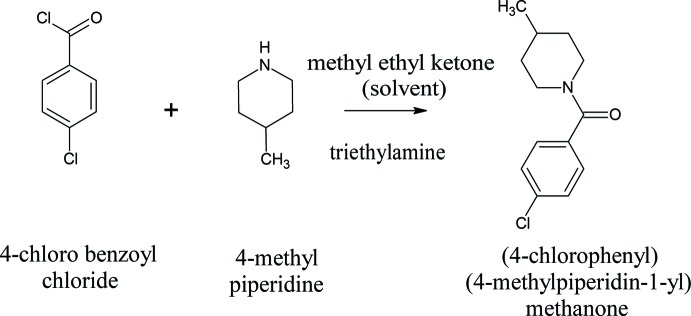
Reaction scheme.

The title compound was synthesized using the published procedure (Revathi *et al.*, 2018 ▸) *via* a Scholten–Boumann condensation reaction (Fig. 5 ▸). A homogeneous mixture of the reagent, 4-methyl­piperidine (0.04mol) was prepared with 150ml of methyl ethyl ketone in a round-bottomed flask by stirring it at room temperature for a few minutes. Then 0.04mol of tri­ethyl­amine was added, followed by stirring for 20min. An equal amount of 2-chloro­benzoyl chloride (0.04mol) was then added slowly under constant stirring and the mixture was then refluxed for 3h at room temperature. The precipitate of tri­ethyl­ammonium chloride formed was filtered off and the filtrate was allowed to evaporate to obtain the title compound. The product was then recrystallized three times from chloro­form to obtain block-like single crystals of the title compound, m.p. 325K.

## Refinement   

Crystal data, data collection and structure refinement details are summarized in Table 2 ▸. H atoms were positioned geometrically (C—H = 0.93–0.97 Å) and refined as riding with *U*_iso_(H) = 1.5*U*_eq_(C-meth­yl) or 1.2*U*_eq_(C) for all other H atoms.

## Supplementary Material

Crystal structure: contains datablock(s) I. DOI: 10.1107/S2056989020001930/jj2221sup1.cif

Structure factors: contains datablock(s) I. DOI: 10.1107/S2056989020001930/jj2221Isup2.hkl

Supporting information file. DOI: 10.1107/S2056989020001930/jj2221Isup3.cml

CCDC reference: 1973841

Additional supporting information:  crystallographic information; 3D view; checkCIF report

## Figures and Tables

**Table 1:** Hydrogen-bond geometry (Å, °) *Cg*2 is the centroid of the C7–C12 ring.

*D*—H⋯*A*	*D*—H	H⋯*A*	*D*⋯*A*	*D*—H⋯*A*
C11—H11⋯O1^i^	0.93	2.46	3.166 (3)	132
C2—H2*A*⋯*Cg*2^ii^	0.97	2.95	3.843 (3)	154

Symmetry codes: (i) 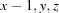; (ii) 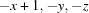.

**Table 2:** Experimental details

Crystal data	
Chemical formula	C_13_H_16_ClNO
*M*_r_	237.72
Crystal system, space group	Triclinic, *P* 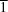
Temperature (K)	296
*a*, *b*, *c* (Å)	6.6286 (4), 8.1569 (5), 12.0061 (8)
α, β, γ (°)	96.803 (3), 101.506 (3), 98.511 (3)
*V* (Å^3^)	621.73 (7)
*Z*	2
Radiation type	Mo *K*α
μ (mm^−1^)	0.29
Crystal size (mm)	0.25 × 0.20 × 0.15

Data collection	
Diffractometer	Bruker Kappa-axis
Absorption correction	Multi-scan (*SADABS*; Bruker, 2012 ▸)
*T*_min_, *T*_max_	0.840, 0.842
No. of measured, independent and observed [*I* > 2σ(*I*)] reflections	11137, 2178, 1687
*R*_int_	0.061
(sin θ/λ)_max_ (Å^−1^)	0.596

Refinement	
*R*[*F* ^2^ > 2σ(*F* ^2^)], *wR*(*F* ^2^), *S*	0.049, 0.144, 1.05
No. of reflections	2178
No. of parameters	147
H-atom treatment	H-atom parameters constrained
Δρ_max_, Δρ_min_ (e Å^−3^)	0.18, −0.25

Computer programs: *APEX3*, *SAINT* and *XPREP* (Bruker, 2012 ▸), *SHELXS97* (Sheldrick, 2008 ▸), *SHELXL2014* (Sheldrick, 2015 ▸), *ORTEP-3 for Windows* (Farrugia, 2012 ▸), *Mercury* (Macrae *et al.*, 2020 ▸) and *publCIF* (Westrip, 2010 ▸).

## supplementary crystallographic information

### Crystal data

**Table:**

C_13_H_16_ClNO	*Z* = 2
*M**_r_* = 237.72	*F*(000) = 252
Triclinic, *P*1	*D*_x_ = 1.270 Mg m^−^^3^
*a* = 6.6286 (4) Å	Mo *K*α radiation, λ = 0.71073 Å
*b* = 8.1569 (5) Å	Cell parameters from 6685 reflections
*c* = 12.0061 (8) Å	θ = 2.9–25.1°
α = 96.803 (3)°	µ = 0.29 mm^−^^1^
β = 101.506 (3)°	*T* = 296 K
γ = 98.511 (3)°	Block, colourless
*V* = 621.73 (7) Å^3^	0.25 × 0.20 × 0.15 mm

### Data collection

**Table:**

Bruker Kappa APEX3 CMOS diffractometer	1687 reflections with *I* > 2σ(*I*)
Radiation source: fine-focus sealed tube	*R*_int_ = 0.061
ω and φ scan	θ_max_ = 25.1°, θ_min_ = 3.3°
Absorption correction: multi-scan (*SADABS*; Bruker, 2012)	*h* = −7→7
*T*_min_ = 0.840, *T*_max_ = 0.842	*k* = −9→9
11137 measured reflections	*l* = −14→14
2178 independent reflections	

### Refinement

**Table:**

Refinement on *F*^2^	Secondary atom site location: difference Fourier map
Least-squares matrix: full	Hydrogen site location: inferred from neighbouring sites
*R*[*F*^2^ > 2σ(*F*^2^)] = 0.049	H-atom parameters constrained
*wR*(*F*^2^) = 0.144	*w* = 1/[σ^2^(*F*_o_^2^) + (0.0575*P*)^2^ + 0.3053*P*] where *P* = (*F*_o_^2^ + 2*F*_c_^2^)/3
*S* = 1.05	(Δ/σ)_max_ < 0.001
2178 reflections	Δρ_max_ = 0.18 e Å^−^^3^
147 parameters	Δρ_min_ = −0.25 e Å^−^^3^
0 restraints	Extinction correction: SHELXL2014 (Sheldrick, 2008), Fc^*^=kFc[1+0.001xFc^2^λ^3^/sin(2θ)]^-1/4^
Primary atom site location: structure-invariant direct methods	Extinction coefficient: 0.108 (19)

### Special details

**Table:**

Geometry. All esds (except the esd in the dihedral angle between two l.s. planes) are estimated using the full covariance matrix. The cell esds are taken into account individually in the estimation of esds in distances, angles and torsion angles; correlations between esds in cell parameters are only used when they are defined by crystal symmetry. An approximate (isotropic) treatment of cell esds is used for estimating esds involving l.s. planes.
Refinement. Refinement of F^2^ against ALL reflections. The weighted R-factor wR and goodness of fit S are based on F^2^, conventional R-factors R are based on F, with F set to zero for negative F^2^. The threshold expression of F^2^ > 2sigma(F^2^) is used only for calculating R-factors(gt) etc. and is not relevant to the choice of reflections for refinement. R-factors based on F^2^ are statistically about twice as large as those based on F, and R- factors based on ALL data will be even larger.

### Fractional atomic coordinates and isotropic or equivalent isotropic displacement parameters (Å^2^)

**Table:**

	*x*	*y*	*z*	*U*_iso_*/*U*_eq_	
Cl1	0.24442 (13)	0.18102 (11)	−0.44227 (6)	0.0859 (4)	
O1	1.1366 (2)	0.3558 (2)	−0.04876 (15)	0.0658 (5)	
N1	0.9452 (3)	0.2267 (2)	0.06264 (17)	0.0550 (5)	
C1	0.7665 (4)	0.1096 (3)	0.0775 (2)	0.0568 (6)	
H1A	0.8106	0.0055	0.0947	0.068*	
H1B	0.6601	0.0848	0.0065	0.068*	
C2	0.6753 (4)	0.1832 (3)	0.1740 (2)	0.0592 (6)	
H2A	0.5635	0.1005	0.1858	0.071*	
H2B	0.6158	0.2795	0.1523	0.071*	
C3	0.8399 (4)	0.2370 (3)	0.2863 (2)	0.0627 (7)	
H3	0.8885	0.1362	0.3104	0.075*	
C4	1.0257 (4)	0.3522 (4)	0.2649 (2)	0.0699 (8)	
H4A	0.9837	0.4571	0.2478	0.084*	
H4B	1.1357	0.3770	0.3343	0.084*	
C5	1.1111 (4)	0.2761 (4)	0.1664 (2)	0.0672 (7)	
H5A	1.2220	0.3572	0.1521	0.081*	
H5B	1.1696	0.1787	0.1869	0.081*	
C6	0.9693 (3)	0.2802 (3)	−0.0362 (2)	0.0511 (6)	
C7	0.7827 (3)	0.2502 (3)	−0.1352 (2)	0.0488 (5)	
C8	0.8006 (4)	0.1799 (3)	−0.2430 (2)	0.0557 (6)	
H8	0.9259	0.1473	−0.2518	0.067*	
C9	0.6368 (4)	0.1573 (3)	−0.3373 (2)	0.0609 (7)	
H9	0.6494	0.1071	−0.4087	0.073*	
C10	0.4536 (4)	0.2104 (3)	−0.3242 (2)	0.0566 (6)	
C11	0.4326 (4)	0.2848 (3)	−0.2191 (2)	0.0551 (6)	
H11	0.3092	0.3221	−0.2118	0.066*	
C12	0.5960 (3)	0.3036 (3)	−0.1249 (2)	0.0521 (6)	
H12	0.5816	0.3525	−0.0535	0.062*	
C13	0.7481 (6)	0.3170 (4)	0.3813 (3)	0.0895 (10)	
H13A	0.6345	0.2386	0.3935	0.134*	
H13B	0.6978	0.4156	0.3593	0.134*	
H13C	0.8543	0.3475	0.4511	0.134*	

### Atomic displacement parameters (Å^2^)

**Table:**

	*U*^11^	*U*^22^	*U*^33^	*U*^12^	*U*^13^	*U*^23^
Cl1	0.0826 (6)	0.1031 (7)	0.0649 (5)	0.0208 (4)	0.0003 (4)	0.0062 (4)
O1	0.0437 (9)	0.0777 (12)	0.0767 (12)	−0.0028 (8)	0.0272 (8)	0.0079 (9)
N1	0.0407 (10)	0.0592 (12)	0.0636 (12)	−0.0009 (8)	0.0120 (9)	0.0143 (9)
C1	0.0479 (13)	0.0535 (13)	0.0664 (15)	−0.0048 (10)	0.0135 (11)	0.0140 (11)
C2	0.0474 (13)	0.0637 (15)	0.0682 (16)	0.0030 (11)	0.0162 (11)	0.0194 (12)
C3	0.0659 (15)	0.0614 (15)	0.0615 (15)	0.0072 (12)	0.0129 (12)	0.0197 (12)
C4	0.0630 (16)	0.0730 (17)	0.0637 (16)	−0.0050 (13)	−0.0014 (12)	0.0171 (13)
C5	0.0413 (12)	0.0772 (18)	0.0788 (18)	−0.0013 (12)	0.0037 (12)	0.0266 (14)
C6	0.0451 (12)	0.0480 (12)	0.0637 (14)	0.0078 (10)	0.0223 (10)	0.0046 (10)
C7	0.0457 (12)	0.0451 (12)	0.0577 (13)	0.0025 (9)	0.0208 (10)	0.0076 (10)
C8	0.0533 (13)	0.0574 (14)	0.0620 (15)	0.0083 (11)	0.0280 (12)	0.0075 (11)
C9	0.0714 (16)	0.0623 (15)	0.0521 (14)	0.0101 (12)	0.0251 (12)	0.0033 (11)
C10	0.0596 (14)	0.0567 (14)	0.0534 (13)	0.0056 (11)	0.0150 (11)	0.0092 (11)
C11	0.0481 (12)	0.0586 (14)	0.0606 (14)	0.0084 (10)	0.0172 (11)	0.0100 (11)
C12	0.0497 (13)	0.0542 (13)	0.0545 (13)	0.0061 (10)	0.0218 (11)	0.0037 (10)
C13	0.104 (2)	0.094 (2)	0.074 (2)	0.0115 (19)	0.0294 (18)	0.0131 (17)

### Geometric parameters (Å, º)

**Table:**

Cl1—C10	1.740 (3)	C5—H5A	0.9700
O1—C6	1.233 (3)	C5—H5B	0.9700
N1—C6	1.343 (3)	C6—C7	1.502 (3)
N1—C5	1.459 (3)	C7—C8	1.388 (3)
N1—C1	1.462 (3)	C7—C12	1.394 (3)
C1—C2	1.513 (3)	C8—C9	1.377 (3)
C1—H1A	0.9700	C8—H8	0.9300
C1—H1B	0.9700	C9—C10	1.379 (4)
C2—C3	1.527 (3)	C9—H9	0.9300
C2—H2A	0.9700	C10—C11	1.376 (3)
C2—H2B	0.9700	C11—C12	1.378 (3)
C3—C4	1.519 (4)	C11—H11	0.9300
C3—C13	1.522 (4)	C12—H12	0.9300
C3—H3	0.9800	C13—H13A	0.9600
C4—C5	1.517 (4)	C13—H13B	0.9600
C4—H4A	0.9700	C13—H13C	0.9600
C4—H4B	0.9700		
			
C6—N1—C5	120.52 (19)	N1—C5—H5B	109.6
C6—N1—C1	125.8 (2)	C4—C5—H5B	109.6
C5—N1—C1	113.55 (19)	H5A—C5—H5B	108.1
N1—C1—C2	110.72 (19)	O1—C6—N1	123.0 (2)
N1—C1—H1A	109.5	O1—C6—C7	118.5 (2)
C2—C1—H1A	109.5	N1—C6—C7	118.53 (19)
N1—C1—H1B	109.5	C8—C7—C12	118.3 (2)
C2—C1—H1B	109.5	C8—C7—C6	119.34 (19)
H1A—C1—H1B	108.1	C12—C7—C6	122.1 (2)
C1—C2—C3	111.9 (2)	C9—C8—C7	121.4 (2)
C1—C2—H2A	109.2	C9—C8—H8	119.3
C3—C2—H2A	109.2	C7—C8—H8	119.3
C1—C2—H2B	109.2	C8—C9—C10	118.9 (2)
C3—C2—H2B	109.2	C8—C9—H9	120.5
H2A—C2—H2B	107.9	C10—C9—H9	120.5
C4—C3—C13	112.4 (2)	C11—C10—C9	121.2 (2)
C4—C3—C2	109.3 (2)	C11—C10—Cl1	119.38 (19)
C13—C3—C2	111.4 (2)	C9—C10—Cl1	119.45 (19)
C4—C3—H3	107.9	C10—C11—C12	119.4 (2)
C13—C3—H3	107.9	C10—C11—H11	120.3
C2—C3—H3	107.9	C12—C11—H11	120.3
C5—C4—C3	112.6 (2)	C11—C12—C7	120.8 (2)
C5—C4—H4A	109.1	C11—C12—H12	119.6
C3—C4—H4A	109.1	C7—C12—H12	119.6
C5—C4—H4B	109.1	C3—C13—H13A	109.5
C3—C4—H4B	109.1	C3—C13—H13B	109.5
H4A—C4—H4B	107.8	H13A—C13—H13B	109.5
N1—C5—C4	110.3 (2)	C3—C13—H13C	109.5
N1—C5—H5A	109.6	H13A—C13—H13C	109.5
C4—C5—H5A	109.6	H13B—C13—H13C	109.5
			
C6—N1—C1—C2	−126.7 (2)	O1—C6—C7—C8	50.7 (3)
C5—N1—C1—C2	57.3 (3)	N1—C6—C7—C8	−130.7 (2)
N1—C1—C2—C3	−55.0 (3)	O1—C6—C7—C12	−123.7 (2)
C1—C2—C3—C4	52.9 (3)	N1—C6—C7—C12	54.8 (3)
C1—C2—C3—C13	177.7 (2)	C12—C7—C8—C9	−2.3 (3)
C13—C3—C4—C5	−177.3 (2)	C6—C7—C8—C9	−176.9 (2)
C2—C3—C4—C5	−53.1 (3)	C7—C8—C9—C10	1.8 (4)
C6—N1—C5—C4	126.9 (2)	C8—C9—C10—C11	0.0 (4)
C1—N1—C5—C4	−56.8 (3)	C8—C9—C10—Cl1	−179.41 (19)
C3—C4—C5—N1	54.8 (3)	C9—C10—C11—C12	−1.3 (4)
C5—N1—C6—O1	8.4 (4)	Cl1—C10—C11—C12	178.09 (18)
C1—N1—C6—O1	−167.4 (2)	C10—C11—C12—C7	0.8 (3)
C5—N1—C6—C7	−170.0 (2)	C8—C7—C12—C11	0.9 (3)
C1—N1—C6—C7	14.2 (3)	C6—C7—C12—C11	175.4 (2)

### Hydrogen-bond geometry (Å, º)

*Cg*2 is the centroid of the C7–C12 ring.

**Table:**

*D*—H···*A*	*D*—H	H···*A*	*D*···*A*	*D*—H···*A*
C11—H11···O1^i^	0.93	2.46	3.166 (3)	132
C2—H2*A*···*Cg*2^ii^	0.97	2.95	3.843 (3)	154

Symmetry codes: (i) *x*−1, *y*, *z*; (ii) −*x*+1, −*y*, −*z*.

### The surface property statistics for the generated Hirshfeld Surface

**Table:**

Surface parameter	Min	Mean	Max
*d*_i_ (Å)	1.030	1.696	2.851
*d*_e_ (Å	1.031	1.699	2.694
*d*_norm_ (Å)	-0.176	0.537	1.463
